# Helper Response to Experimentally Manipulated Predation Risk in the Cooperatively Breeding Cichlid *Neolamprologus pulcher*

**DOI:** 10.1371/journal.pone.0010784

**Published:** 2010-05-26

**Authors:** Dik Heg, Michael Taborsky

**Affiliations:** Department of Behavioural Ecology, Institute of Ecology and Evolution, University of Bern, Hinterkappelen, Switzerland; Vrije Universiteit, Netherlands

## Abstract

**Background:**

We manipulated predation risk in a field experiment with the cooperatively breeding cichlid *Neolamprologus pulcher* by releasing no predator, a medium- or a large-sized fish predator inside underwater cages enclosing two to three natural groups. We assessed whether helpers changed their helping behaviour, and whether within-group conflict changed, depending on these treatments, testing three hypotheses: ‘pay-to-stay’ PS, ‘risk avoidance’ RA, or (future) reproductive benefits RB. We also assessed whether helper food intake was reduced under risk, because this might reduce investments in other behaviours to save energy.

**Methodology/Principal Findings:**

Medium and large helpers fed less under predation risk. Despite this effect helpers invested more in territory defence, but not territory maintenance, under the risk of predation (supporting PS). Experimentally covering only the breeding shelter with sand induced more helper digging under predation risk compared to the control treatment (supporting PS). Aggression towards the introduced predator did not differ between the two predator treatments and increased with group member size and group size (supporting PS and RA). Large helpers increased their help ratio (helping effort/breeder aggression received, ‘punishment’ by the dominant pair in the group) in the predation treatments compared to the control treatment, suggesting they were more willing to PS. Medium helpers did not show such effects. Large helpers also showed a higher submission ratio (submission/ breeder aggression received) in all treatments, compared to the medium helpers (supporting PS).

**Conclusions/Significance:**

We conclude that predation risk reduces helper food intake, but despite this effect, helpers were more willing to support the breeders, supporting PS. Effects of breeder punishment suggests that PS might be more important for large compared to the medium helpers. Evidence for RA was also detected. Finally, the results were inconsistent with RB.

## Introduction

Avoidance of predators is thought to be an important benefit of group living (see reviews [1]–[3]). Within groups, animals may benefit from the presence and protection of the other group members (e.g. [4]–[11]), but these benefits must outweigh the costs of group-living due to e.g. groups attracting more predators (e.g. [12]) or within-group competition over access to resources (e.g. [13], [14]). Predation risk has been proposed to increase the net benefits of remaining in their (natal) group for subordinates of cooperatively breeding species (‘ecological constraints hypothesis’, [11], [15]). These benefits may be especially high in large groups due to e.g., being protected from predation by the larger group members or risk dilution effects (‘group augmentation hypothesis’, [11], [16], [17]).

The purpose of the present paper is to test effects of experimentally varied risk of predation on helping behaviour and within-group conflict in the cichlid *Neolamprologus pulcher*. *N. pulcher* has been a model species for testing hypotheses proposed to explain when subordinate group members should engage in reproduction (so called ‘helpers’, [18]–[22]), disperse [11], and ‘pay-to-stay’ [23], [24] for group membership [25]–[29]. Support has been found for the ‘ecological constraints’ (e.g. helpers delay dispersal under predation risk [11] and preferentially disperse to new shelters within the colony [30]), ‘group augmentation’ (e.g. helping may accrue benefits to all group members due to survival increasing with group size [11], [17], [31]) and ‘pay-to-stay’ hypotheses (e.g. helping and appeasement behaviour is tailored to previous helper investment, demand and opportunities to disperse [25]–[29], [32] or tailored to increase subordinate reproduction [33]) in a large variety of experimental field and laboratory studies. In a field experiment we introduced medium or large-sized predators to large underwater cages encompassing two or three *N. pulcher* group-territories. In similar control cages no predators were introduced. In a previous paper, we analysed the effects of the treatments on helper survival, helper dispersal and group reproductive success [11]. We concluded that under the risk of predation, group membership became more valuable to the helpers whereas independent breeding became less valuable and more risky, and therefore helper dispersal was reduced. Here we test effects of the treatments on helper food intake, helping behaviour (separate for territory maintenance, which is digging and sand carrying combined; and territory defence) and breeder appeasement. We acknowledge that it is very difficult to derive testable predictions, particularly because the fitness effects of each single helping behaviour has not been tested in any cooperatively breeding fish species (both for the actor and the recipient). This makes the adaptive interpretation of any changes in helping behaviour difficult (see [34]). Nevertheless, we know that all helping and submissive behaviours are energetically expensive in our study species [35], [36], helpers do increase breeder fitness [4], [11], [18] and helpers can release breeders from their duties [22], [31], [33], [37]–[38]. In the following we carefully expose our predictions and leave it to the reader to critically reappraise or re-interpret them, in the light of our results.

We start with testing how the predator treatments affected helper food intake and whether differences may have been mediated by treatment effects on spacing behaviour [11]. If helpers have a reduced food intake under predation risk, this will reduce their body reserves and therefore might reduce their investment in helping behaviours and breeder appeasement. These potential effects have to be taken into account when discussing the forthcoming predictions tested.

In the next part we test three hypotheses regarding the effects of predation risk on helper effort. (1) If helpers ‘pay-to-stay’, they should increase their helping effort (both digging and territory defence) under the risk of predation to reduce the incentives of expulsion by the breeders, compared to the control treatment. Helpers are particularly likely to increase their effort, because prolonged group membership has more effects on helper survival than breeder survival under the risk of predation [11]. Helpers tended to survive less under the large predator treatment compared to the medium predator treatment [11], so if these two treatments differ, we expected a greater change in helping effort in the large predator treatment, particularly for the large helpers which are exposed to a greater expulsion risk compared to the medium helpers [20], [32].

(2) If helpers tailor their helping effort to avoid immediate predation (‘risk avoidance hypothesis’), firstly, risky helping behaviours like territory defence should be reduced if immediate helper survival interests prevail (survival was reduced under predation [11]). Territory defence in cichlids involves leaving the protection of the shelter to attack fish [39], [40], large group members carry bite marks from doing so [31] and piscivores do try to grab and eat cichlids leaving their shelters (D. Heg, personal observations). Secondly, helpers should invest more into territory maintenance (i.e. digging away sand from shelters) in the predator treatments compared to the control treatment, since more and better shelters to hide in are crucial for survival with predators, but not in the control treatment. Thirdly, helpers should not attack the introduced predator at all, since they pose an immediate threat on helper survival. All three effects (reduction in territory defence or introduced predator defence, and increased investment in digging) should be particularly strong in the medium helpers compared to the large helpers, since their survival was more reduced compared to the large helpers in the presence of predators[11]. These predictions run opposite to the predictions made in (1), except for digging behaviour (general increase in both hypotheses).

(3) If helpers tailor their helping effort to their direct reproductive benefits through reproductive participation [20]–[21], [33], [37]–[38] or to the expected future fitness benefits by inheriting the territory [25], [41], [42], all helping behaviours should be reduced in the predator treatments compared to the control treatment. This is because group reproductive success was virtually reduced to zero in the predator treatments [11], and mortality of the helpers was substantially more increased than the mortality of the breeders in the predator treatments compared to the control treatment [11], making inheritance unlikely. We can predict the same effect if the level of help is positively related to the number of offspring present in the group (kin selection [28], [41] or helper reproductive participation leading to helpers willing to invest more when own offspring are present, particularly for the female helpers [33], [37]–[38]), because reproduction under predation risk was very low [11] so no kin or own offspring will be present as recipients of the helping behaviour. Large helpers are more likely to engage in reproduction [21], [33], [37]–[38] or inherit the territory [25], [42] compared to medium helpers, so the reduction in all helping behaviours in the predator treatments compared to the control treatment, should be more pronounced in the large helpers compared to the medium helpers. These predictions are similar to those made for (2), except that we now expect all helping behaviours to decrease in the predator treatments, and the expected difference between large and medium helpers runs opposite from (2). Note that we did not explicitly generate predictions for the group augmentation hypothesis [16], as it may predict both increased helping under predation risk (similar to (1): to ensure continued group membership and thereby future inheritance) and decreased helping under predation risk (similar to (3): the inheritance probability and reproductive success is anyway low, so helping should be reduced).

In the final part, we tested the same three hypotheses regarding the effects of predation risk on within-group interactions. (1) If group members are more valuable under the risk of predation because they pay more, within-group conflict, breeder-helper conflict and breeder punishment of helpers [43], [44] should be reduced in the predator treatments compared to the control treatment. In contrast, friendly within-group social contacts should show the reverse. Note however that a similar result may appear if group members are somehow more valuable irrespective of their level of help provided (e.g. due to dilution effects on predation risk). If group members assess how valuable other group members are based on their investment in helping, (2) the level of within-group conflict, breeder-helper conflict and breeder punishment of helpers should depend on the level of help the subordinates provide (negative correlation), but maybe modified also by appeasement behaviour (submissiveness) shown by the helpers towards the dominant pair [26]. Depending on these relationships, conflicts may not change, increase or decrease under risk avoidance, because helpers should invest more in digging (which could potentially benefit all group members as it creates hiding shelters), but this is offset with a reduced investment in the other helping behaviours (e.g. territory defence) and might be also offset by increased submissiveness. In contrast, (3) if helpers in general help less overall, because they are unlikely to gain direct reproductive benefits and inherit the territory under predation risk, within-group conflict, breeder-helper conflict and breeder punishment of helpers should increase in the predator treatments compared to the control treatment.

Helpers can potentially increase two types of behaviours to reduce the level of conflict with breeders (‘appeasement’ [26]): helping behaviour (i.e. territory defence, digging and brood care) or submissive behaviour (i.e. tail-quivering and zig-zag swimming). Breeder punishment is defined as breeders attacking their group member helper. To test for the effects of the treatments on breeder-helper conflict and punishment independent from the level of help provided, we tested for effects of the treatments on help ratio (help divided by breeder aggression received) and submission ratio (submission divided by breeder aggression received). If (1) applies one or both of these ratios should be higher in the predator treatments compared to the control treatment, if (2) applies we expect no change, if (3) applies one or both of these ratios should be lower in the predator treatments compared to the control treatment.

Additional to the focal helper observations, we performed a standardised experimental helping test in one year only (covering the breeding shelter partly with sand and measure digging behaviour of each group member) to be better able to distinguish between (1), (2) and (3). We also measured aggression towards the introduced predators for all group members alongside the focal helper observations, to assess which group members were willing to invest in such risky defence, and to assess how risk might be diluted due to joint predator defence. Specifically, we expect helpers not to attack the introduced predators if they (2) show risk avoidance or (3) target reproductive/inheritance benefits, but they might show attacks if they (1) pay-to-stay.

## Materials and Methods

### Ethics statement

Predators occur at very high densities in this colony (mean per 10 m^2^±s.d. from [30]: 15.8±25.8, range: 2.7–106.4, *n* = 16) and hunt their fish prey by moving through the colony, often in groups (own observations). The experiment amounted to removing all medium and large predators from the system, hence reducing the impact of group hunting and the impact of certain predator species specializing on either offspring or adults. By reintroducing only one medium or large predator to the experimental treatments we created a moderate level of predation pressure (1 predator per 4 m^2^ in the cage vs. 6.3 per 4 m^2^ from [30]). All fish living inside the cages were monitored every three to five days for signs of stress and well-being. The *N. pulcher* accepted the cages, no individual persistently attempted to escape from the cage, and all fish showed normal feeding behaviour (see Results). Five predators were released from the cages during the experiment and replaced with a similar sized predator because they did not adapt to the cage and showed signs of stress, i.e. they persistently tried to escape, did not show their normal stalking behaviour or stayed motionless all the time. Additionally, all predators were fed pieces of dead fish once every three to five days, to make sure they remained well fed, and to make sure they did not need to rely heavily upon preying on *N. pulcher* to cover their energy budget. Therefore, the predator treatments induced a predation risk, but risk was not exaggerated due to starving predators needing to feed on fish inside the cage to remain viable. The experiment described in this manuscript complies with the current laws of Zambia, the country in which the study was conducted, and was approved by the Zambia Ministry of Agriculture, Food and Fisheries.

### Study species

*N. pulcher* lives in ‘extended family groups’, i.e. a dominant breeding pair with up to 14 subordinates defending a territory and their offspring [17], [31], [32], [39], [45]. Group members deter predators by dashes, ramming and biting. Group reproductive success and group persistence increases with group size [17], [31] and experimental removal of subordinates decreases the productivity and reproductive success of the group [4], [18]. Reproductive skew within groups is high, with the large dominant pair siring the majority of offspring [21], [33], [37]–[38], [46], [47]. Helping is costly, both in terms of time and energy [35], [36] and growth rate [4], [27], [48]. Combining these facts, we conclude subordinates provide active, costly help and therefore they are usually called ‘helpers’. Nevertheless, helpers also impose costs on the breeders, because large mature male helpers engage in parasitic spawning [20]–[22], [49], large mature female helpers may try to breed with the breeder male [33], [37]–[38] also in a separate breeding shelter [38], which may draw away breeder male or helper assistance from the primary female's brood [45], and both helper males and females may compete for the breeding position with the breeder male and female, respectively [25]. These effects are also apparent in sex-dependent dominant-subordinate interactions [50].

### Study site

We studied *N. pulcher* by SCUBA diving at the south tip of Lake Tanganyika, at Kasakalawe point near Mpulungu, Zambia (8°46.849′ S, 31°04.882′ E) from 5 March to 27 May 2002 and 2 February to 21 April 2003. The study population consists of several, partly connected colonies at 9.0 to 11.5 m depth in a sandy area with rocks half submerged in the sand [30]. The present experiments were conducted at colony 2 (*>*200 groups in three sub-patches [30]). In this colony, *N. pulcher* groups breed in distinct patches of stones, and shelters are maintained and extended between and underneath these stones by digging away sand. Groups create a breeding shelter for the breeders (where eggs are laid on the stone surface) and hiding shelters for all group members [31], [51]. Breeders, large and medium-sized helpers preferably forage in the water column (50 to 100 cm above the substrate), where zooplankton is most abundant [52], but retreat to the breeding and hiding shelters as soon as piscivore predators appear.

### Experimental set-up

To prevent pseudo-replication and carry-over effects, different groups were used in the 2002 and 2003 experiments. Experimental units were created as follows. Two to three nearby groups, within 1 to 1.5 m distance were selected haphazardly. All group-territories were marked with numbered rocks and group composition was determined (number and size of breeding males, females, helpers and free swimming fry). Two to four helpers per group were captured by directing them with hand-nets into a plexiglass tube, individually marked and their body measurements were taken (standard length SL in mm, 0.5 mm accuracy). Marking involved injecting non-toxic acrylic paint into scale pouches and fin clips of the dorsal and anal fins. Other group members were recognisable from estimates of their size relative to the marked helpers, and natural body markings. SL was estimated by placing a millimetre board in the territory (0.5 mm classes), and was calibrated to true SL using marked and measured individuals. All marked fish were reaccepted into their respective groups and marked fish showed no signs of adverse effects from the marking method.

A 2 m ×2 m ×2 m cage was put over these two or three groups, removing all predators above 8 cm SL, but including all other naturally occurring fish. Cages consisted of a light-weight aluminium outer frame, all sides except the bottom covered with a sturdy plastic netting (i0.5 mm wire) with inner mesh size 2.5×2.5 mm (Lanz-Anliker AG, Rohrbach, Switzerland), allowing free flow of zooplankton, the main food of *N. pulcher*. The bottom edges on the outside of the cages were covered with flexible nets and rocks, so no fish could enter or leave the cages. Fish inside the cages showed no signs of stress. Per trial, three cages were erected nearby (between cage distance 1 to 5 m). In each trial (*n* = 7, 21 cages in total) one cage was selected at random for the control treatment (no predator added), one cage received the medium predator treatment (one medium sized piscivore *Lepidiolamprologus elongatus* added of SL 11.9±1.6 cm mean ± s.d., range  = 9.9–14.2 cm, *n* = 7) and one cage received the large predator treatment (one large sized *L. elongatus* or *Lamprologus lemairii* added of SL 14.7±1.9 cm mean ± s.d., range = 13.0–17.7 cm, *n* = 7, see [11] for details; in all trials medium predators were smaller than the large predators and there were no significant effects of predator species on the results). Predators were caught by directing them with hand-nets into gill-nets or pouch-nets, catching was done in the immediate vicinity of the cages and predators were immediately transferred to their cage. Trials lasted four weeks, cages were removed and predators released after the end of each trial.

### Focal behavioural observations

Heg and others [11] provides details of the compositions of groups involved in the experiment. Helper behaviour was determined by 15 minute continuous focal observations, using a waterproof stopwatch (all recordings were done by D.H.). Two groups per cage were selected (*n* = 2 groups ×21 cages = 42 groups), and in each group we observed 3 times a medium helper (25.5–35 mm SL) and 3 times a large helper (>35 mm SL), selecting different individuals each time (ca. 5 days between observations, each group alternately). Recording of the medium helper was directly followed by recording of the large helper from the same group, or reverse, in randomised order. To correct for time of day effects [25], [52] and to allow for the best comparison between the treatments possible, three cages from one trial were observed subsequently in one dive, in randomised order. The data from one observation was lost underwater, giving a total sample size of 251 observations (3 observations ×2 helper sizes ×2 groups ×21 cages = 252, minus 1).

The following behavioural parameters were recorded: (1) estimated distance to the nearest shelter in cm (determined every minute, the 15 values averaged - arithmetic mean - per observation before analyses). (2) The total time (seconds) hiding inside a shelter (breeding or other shelter: see [11]). (3) Total food uptake (bites per 15 min observation time) and feeding rate (number of bites per minute not hiding inside shelter). (4) Frequency of carrying sand away in the mouth from the shelter or digging sand away by tail-beating (abbreviated ‘digging’ throughout). (5) Frequency of territory defence (excluding defence against the introduced predator). (6) Within-group aggression: includes restrained and overt aggression directed towards and received from group members. (7) Within-group social contacts: includes submissive displays and bumping towards and from group members received. For all aggressive and social behaviours the recipient of the behaviour was recorded as well (see for detailed descriptions of all behavioural displays: [4], [32], [40], [53]; and particularly [54]). Attacks on the introduced predator were analysed separately (see below). The frequencies of focal behaviours were corrected for the minute the focal individual was not hiding (entered as a fixed covariate see below, cf. [11]). The focal observations were used to test for effects of the treatments on (1) feeding, helping behaviour (separate for digging and territory defence), within-group aggression and within-group social contacts, and (2) help ratio  = ([square-root of digging plus territory defence +3/8] per minute not hiding/([square-root of aggression received from breeders +3/8] per minute not hiding) and submission ratio  = ([square-root of submissive to breeders +3/8] per minute not hiding/([square-root of aggression received from breeders +3/8] per minute not hiding).

### Digging experiment

In 2003 we conducted a digging experiment in two groups per cage (number of groups observed: 2 groups ×1 cage ×3 treatments ×4 trials = 24; only one experiment per group). The purpose was to experimentally generate a standardised need to help, which should give clearer treatment effects than the digging behaviour recorded in the focal observations. The breeding shelter of each group in turn was approximately half covered with sand and immediately followed by a 10 minute observation. In case the group had multiple breeding females each with their own breeding shelter, all breeding shelters were partly covered with sand. During each observation, all events of digging sand away from the experimentally sand-covered breeding shelter(s) by each group member were scored and analysed on a per capita basis per type of group member, i.e. small helper (15.5–25 mm SL), medium helper (25.5–35 mm SL), large helper (>35 mm SL), breeder female, and breeder male. As in one group the breeding male and female already died before this recording, and in another group no small helpers were present, the total sample size was 24 observed groups ×5 types of group members  = 120, minus 3 missing data  = 117.

### Aggression towards the introduced predators

To assess whether and which group members engaged in territory defence against the introduced predators (*n* = 17 groups with medium predator and 16 groups with large predator), the frequency of overt and restrained attacks on these predators for each type of group member were recorded alongside the 15 minute focal observations in the predator treatments. Data were collected in both years. Again, investment in territory defence were scored and analysed on a per capita basis per type of group member, i.e. small helper, medium helper, large helper, breeder female, and breeder male. Sample sizes varied because not all types of group members were present or still alive in the focal groups on the day of observation, but a total *n* = 803 observations were available (i.e. multiple observations of multiple group members).

### Statistical analyses

The majority of analyses were performed with generalised estimating equations (GEE) in SPSS 17.0. This procedure uses the Restricted Maximum Likelihood Method (REML) to decompose variances and allows incorporation of fixed and random effects. Group identities were entered subjects in all GEEs, to account for repeated measures per group [55]. Effects of our treatment on helping and social behaviours may be mediated or modified by two routes. First, predators forced both medium and large helpers to stay closer to protective shelter and hide more [11], and this might e.g. increase the level of within-group interactions and e.g. decrease the level of aggressive interactions with neighbouring groups [51]. Second, the group composition might affect the level of helping and social behaviours [49], [50]. To test for both these effects, we first constructed GEEs with treatment, helper size and their interaction as independent variables; helper feeding rate and total food intake (normal distributions) or helper behaviour (digging, territory defence, within-group aggression or within-group social contacts; all frequencies with poisson distributions and log-link) were the dependent variables in each GEE (*n* = 50 groups). Time spent hiding was entered as a covariate for helper behaviour, to account for the fish being out of sight. We expected a significant effect of treatment (up or down-regulation of helper behaviour compared to the control treatment, direction depending on the hypothesis) and/or significant effect of the interaction (larger helpers should change more than medium helpers). Next, we added the following two covariates as independent fixed effects to these GEEs: (1) the mean focal individual's distance from protective shelter and (2) the number of adults (breeders plus large helpers >35mm SL) living in the focal's group. This allowed us to assess whether the treatment effects might have been mediated by effects on the focal's mean distance from shelter, or modified by the number of adults in the group. The scaling parameter was adjusted using the deviance method in each GEE.

The effort spent in digging plus sand carrying during the digging experiment was also analysed using a GEE with groups as subjects (*n* = 24 groups, each group tested once only). This digging effort was measured for small, medium, large helpers, breeder females and breeder males separately (so called ‘status’), and we counted the number of individuals observed per status. We tested for the fixed effects of treatment, status and their interaction on digging (poisson distribution, log-link, scaling parameter adjusted using the deviance method), entering the number of individuals observed as a covariate, to arrive at per capita effects. Similarly, defence against the introduced predator was analysed using a GEE with groups as subjects (*n* = 33 groups, each group observed alongside a focal observation). Again, predator attacks were counted per status, and we counted the number of individuals observed per status. We tested for the fixed effects of treatment (medium or large predator), status and their interaction on attacks (poisson distribution, log-link, scaling parameter adjusted using the deviance method), entering the number of individuals observed as a covariate, to arrive at per capita effects.

The help ratio (normal distribution) and submission ratio (gamma distribution with the canonical link power(-1), see [55]) were analysed with GEE (*n* = 50 groups). One observation of a medium helper from the large predator treatment had to be excluded, because the fish was hiding 100% of the time. We entered the fixed effects of treatment, helper size and their interaction.

Total food intake rate and feeding rate per minute were additionally analysed with a two-parameter (a, b) hyperbolic function for medium and large focal helpers separately: feeding rate  =  distance/(a+b× distance), using non-linear regression [55]. This function has as intercept y = x = 0 (i.e. when the helper was hiding, distance was zero, so it could not feed by default), and allows for an exponentially diminishing increase in feeding rate with distance (i.e. maximum feeding rate was constrained, since feeding rate cannot increase indefinitely by default). All significance thresholds were set at alpha  = 0.05.

## Results

### Focal helper feeding behaviour

**Figure 1:**
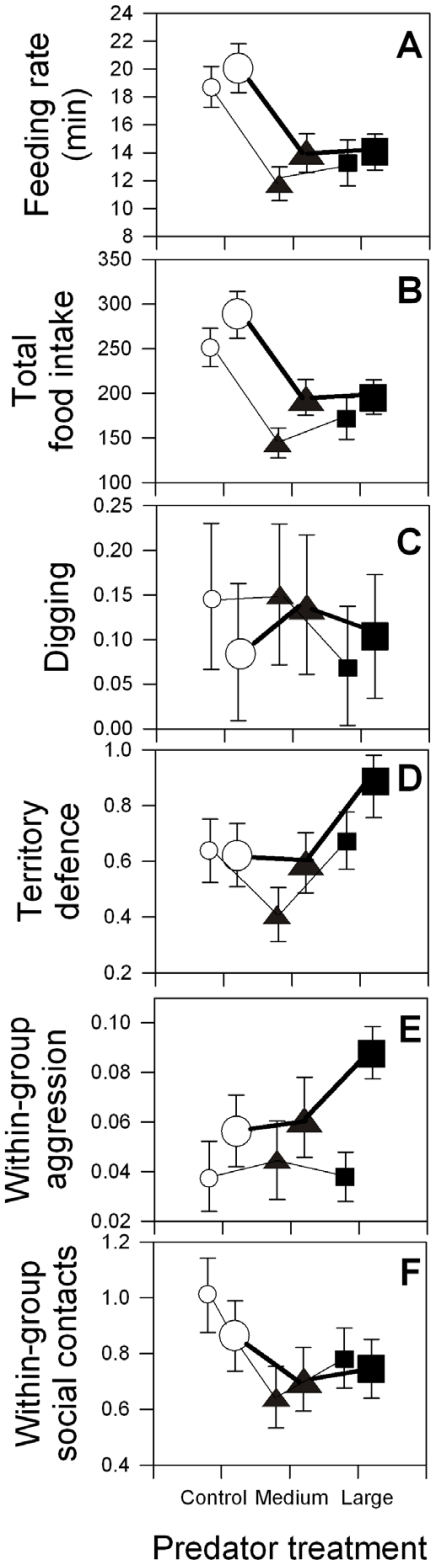
Focal subordinate behaviour depending on the predator treatments. Depicted are means ± s.e.m. per 15 minutes observation time, except (A) per minute not hiding, of behaviour depending on the treatments (white circles: control; black triangles: medium predator; black squares: large predator) and helper size (small symbols and thin lines: medium helpers; large symbols and bold lines: large helpers). For statistics see Table S1. (A) Feeding rate per minute not hiding, (B) total food intake, (C) digging frequency, (D) territory defence frequency (excluding against introduced predator), (E) within-group conflicts, (F) within-group social contacts. Sample sizes are *n* = 42 for each symbol, except for medium helpers large predator treatment (*n* = 41 due to one sample lost).

**Figure 2:**
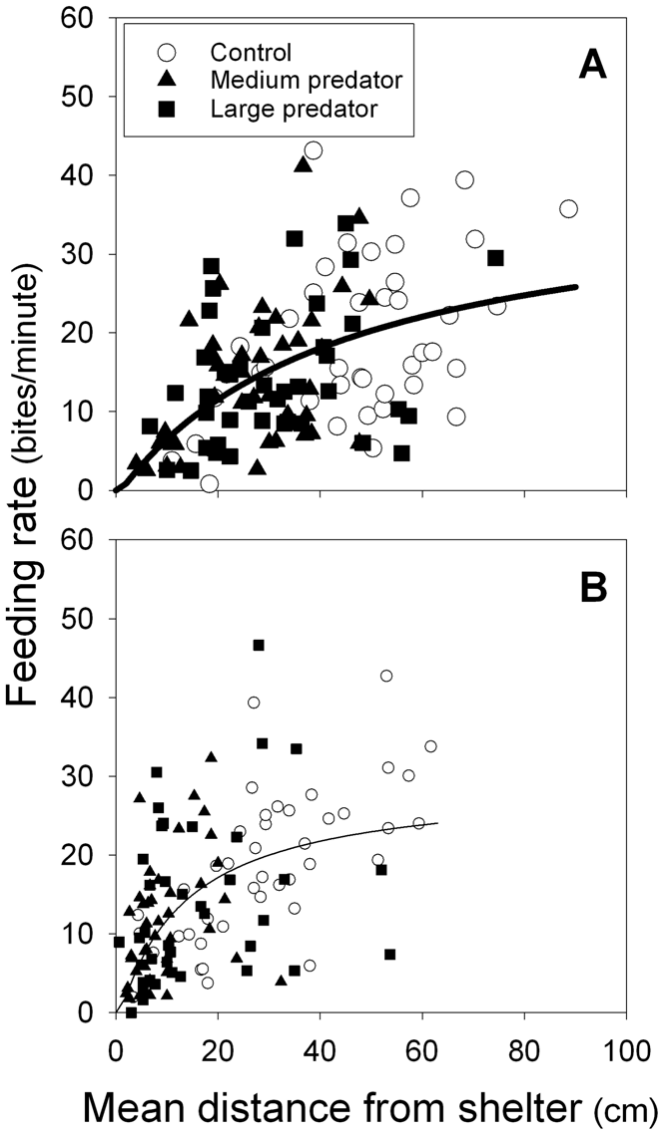
Focal subordinate feeding rate was closely related to spacing behaviour. Intake rate increased with average distance moved from protective shelter in both (A) large helpers and (B) medium helpers. Depicted are hyperbolic curve fits (distance/[a+b x distance]) from non-linear regressions for large helpers (*n* = 126, coefficients ± s.e.): a = 1.1572±0.2642, b = 0.0257±0.0064 (*F*_2,124_ = 229.7, *p*<0.001, *R* ^2^ = 0.23); and for medium helpers (*n* = 125): a = 0.4575±0.0910, b = 0.0342±0.0042 (*F*_2,123_ = 226.6, *p*<0.001, *R* ^2^ = 0.30).

Helpers had a significantly lower feeding rate (Fig. 1a) and total food intake (Fig. 1b) in both predator treatments, compared to the control treatment (Table S1). This effect resulted from helpers feeding closer to the shelters, where the feeding rate appeared lower (Fig. 2, Table S1). Note that corrected for distance, medium helpers (Fig. 2a) had a higher feeding rate per minute than large helpers (Fig. 2b, on average 5.1 more bites per minute ±1.3 s.e., parameter estimate from Table S1 GEE). Nevertheless, the total food intake was significantly lower for the medium compared to the larger helpers (Fig. 1b), because medium helpers were hiding more[11] and fed on average closer to protective shelter than the large helpers (Fig. 2, Table S1). Therefore, subsequent predator-induced negative effects on helper behaviour have to be interpreted with care, since due to their reduced food intake, helpers in the predator treatments may need to save energy by investing less in helping and submissive behaviours.

### Focal helping behaviour

Contrary to our expectations (1) to (3), treatment and interaction (treatment x helper size) effects on digging were non-significant (Fig. 1c, Table S1, GEE left column). These results did not change when the distance to the shelter and the number of adults protecting the group were added to the analyses (Table S1, GEE right column), although focal helpers staying closer to the group were digging significantly harder (*p* = 0.046, Table S1, coefficient ± s.e. of ln[distance]: −0.645±0.323). However, as expected only by (1) the pay to stay hypothesis, treatment significantly affected territory defence (*p* = 0.033, Fig. 1d), but only when corrected for distance (Table S1, GEE right column, coefficient ± s.e. of ln[distance]: 0.592±0.125) and no effects of helper size and the interaction between helper size and treatment were detected in both models.

### Digging experiment

**Figure 3:**
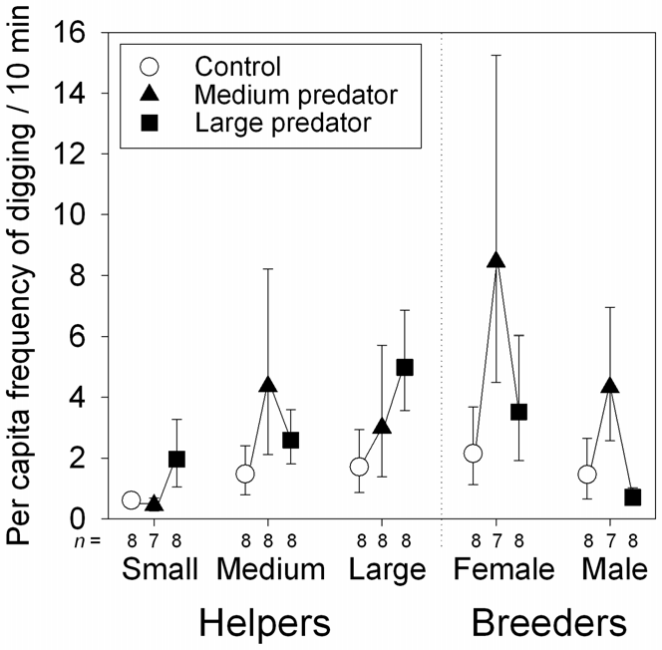
Digging experiment. Effect of covering the breeding shelter with sand on subsequent digging and carrying sand behaviour from this shelter by the different group members in the three predator treatments. Depicted are means ± s.e.m. and sample sizes (number of groups). For statistics see Table 1.

We created also an artificial need for help by covering the breeding shelter with sand and subsequently measured the digging effort of all group members. As expected by both (1) the pay-to-stay and (2) the risk avoidance hypothesis, digging effort increased in the predator treatments compared to the control treatment (Fig. 3), but the effect depended on social status as well (Table 1). This was due to some group members reacting more strongly to the medium predator than to the large predator (compared to the control treatment: medium helpers, and both breeders), whereas others reacted more strongly to the large predator (small and large helpers). Restricting our analysis to the helpers only did not change these results (*n* = 71 of 24 groups: GEE effect of helper size: 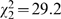, *p*<0.001; treatment: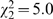, *p* = 0.084; interaction: 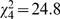, *p*<0.001).

**Table 1:** Digging experiment and attacks on the introduced predator: results of two poisson GEEs with log-link, testing for fixed effects of the treatment, status and their interaction on the digging plus sand carrying effort after the breeding shelter was covered with sand (*n* = 117) and attacks on the introduced predator (*n* = 664) separately.

	Digging effort^a^			Attack rate^b^		
Independent variables	χ^2^	df	*p*	χ^2^	df	*p*
Treatment	5.4	2	0.068	2.0	1	0.16
Status	31.1	4	<0.001	87.5	3	<0.001
Treatment*status	64.3	8	<0.001	2.4	3	0.49
Number of individuals	5.4	1	0.02	0.18	1	0.67

Note that for practical reasons effort was measured for all individuals per status lumped, so the number of individuals observed per status was entered as a covariate to arrive at per capita estimates.

Results were corrected for random group effects (repeated measures of 24 groups for digging and 33 groups for attacks, entered as subjects in the GEEs), the scaling parameter was adjusted using the deviance method in each model.

a Group member status was divided in five classes (*n* = 24 in each case): small helper (15.5–25.0 mm SL, mean individuals per group ± s.d., range: 2.58±1.35, 0–6), medium helper (25.5–35 mm SL, 2.33±1.58, 1–8), large helper (>35 mm SL, 2.33±1.17, 1–6), breeder female (1.04±0.36, 0–2) or breeder male (0.96±0.20, 0–1), so in total 222 individuals were observed. Small helpers were missing for one group with a medium predator, and both the breeder female and breeder male were missing from another group with a medium predator.

b Group member status was divided in five classes (*n* = 33 in each case): small helper (15.5–25.0 mm SL, mean individuals per group ± s.d., range: 2.74±2.03, 1–10), medium helper (25.5–35 mm SL, 2.45±1.58, 1–8), large helper (>35 mm SL, 4.88±2.83, 2–17), breeder female (1.09±0.29, 1–2) or breeder male (1.00±0.00, 1–1), and since these groups were observed multiply we had 803 cases in total. However, since small helpers were never seen to attack the introduced predators, they were omitted from the GEE and this reduced the sample size to 664 (*n* = 33 groups with 8 to 36 measurements per group).

### Defence against the introduced predator

**Figure 4:**
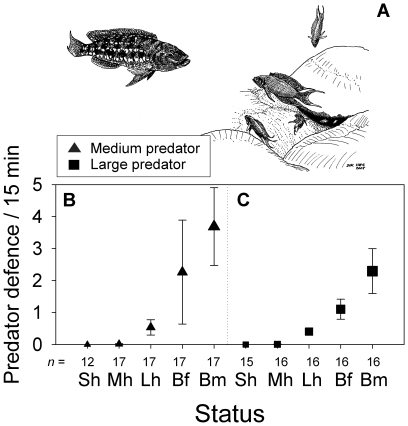
The per capita frequency of aggression against the introduced predator. (A) Shows the introduced predator on the left (*L. elongatus*) and defending group members of *N. pulcher* on the right. Depicted are means ± s.e.m. of aggression and sample sizes (number of groups) for the five different types of groups members in the (B) medium and (C) large predator treatment (Sh: Small helpers, Mh: Medium helpers, Lh: Large helpers, Bf: breeding females, Bm: breeding males). Note that small helpers were never seen to attack the medium and large predators (see text). For statistics see Table 1.

In total 1239 aggressive displays and attacks were observed against the predators (Fig. 4a): 0 by the small helpers, 4 by the medium helpers, 366 by the large helpers, 325 by the breeder females, and 544 by the breeder males. Since small helpers were never seen to attack, or display against, the introduced medium and large predators, they were removed from the subsequent GEE analysis. Medium predators (Fig. 4a) were not treated with more or less aggression than large predators (Fig. 4b, Table 1), but aggression towards both predators significantly increased from medium, large helpers, to breeder female, to breeder male (Fig. 4, Table 1).

Joint defence of the group against the predator was common, resulting in positive correlations between the per capita attack rates of large helpers, breeder females and breeder males, both against the medium predator and the large predator (Table 2). Moreover, the per capita attack rate increased for all group members with the number of adults contained in a group in the large predator treatment, but not in the medium predator treatment (Table 2).

**Table 2:** Results of Spearman Rank Correlations between the per capita frequency of aggression against the predators by the different group members and the number of adults living in the group (number of breeders and large helpers), for the medium (above diagonal) and large predator treatments (below diagonal), separately.

Variable	Number of adults	Aggression by:		
		Large helpers	Breeder females	Breeder males
Number of adults	-	0.15 (84)	−0.06 (84)	0.04 (80)
Aggression by large helpers	0.22* (83)	-	0.50** (84)	0.66** (80)
Aggression by breeder females	0.24* (83)	0.57** (83)	-	0.66** (80)
Aggression by breeder males	0.23* (83)	0.72** (83)	0.83** (83)	-

In brackets sample sizes.

*p<0.05,

**p<0.001.

### Focal helper within-group interactions

**Figure 5:**
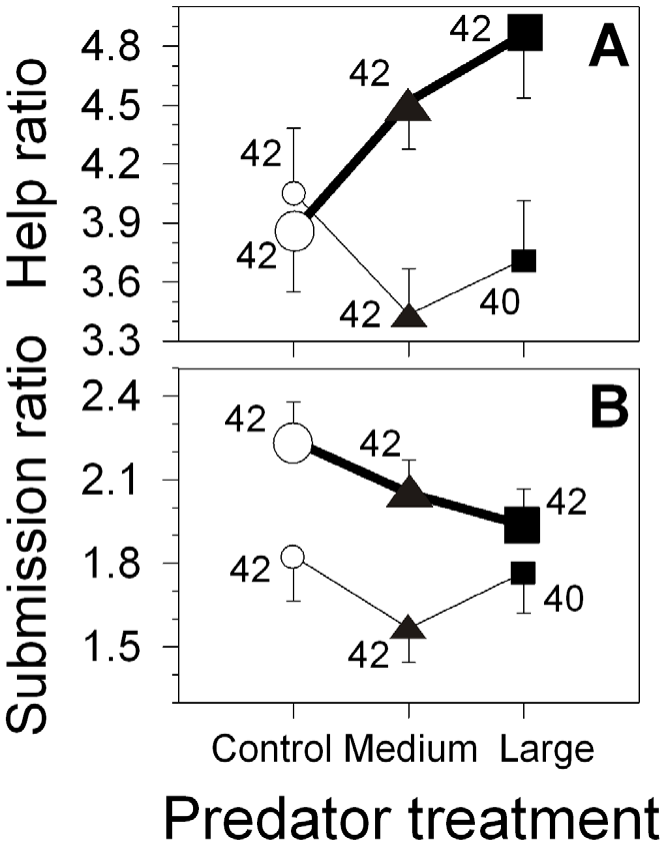
Helper-breeder conflict. The ratio of (A) helping effort and (B) submission shown to the breeders, per received aggression from the breeders depending on the predator treatments (white circles: control; black triangles: medium predator; black squares: large predator) and helper size (small symbols and thin lines: medium helpers; large symbols and bold lines: large helpers). Depicted are means ± s.e.m (ratios =  [frequency helping or submission square-root +3/8 transformed]/[frequency breeder aggression received square-root +3/8 transformed]) with sample sizes (number of observations). For statistics see text. Note that the sample size for medium helpers in the large predator treatment was *n* = 40 due to one sample lost and one sample the helper was hiding 100% of the time (gives 42–2 = 40).

There were no differences between the treatments regarding within-group aggression (Fig. 1e) and within-group social contacts (Fig. 1f, Table S1); and workload (digging and territory defence combined) and submissive behaviours to the breeders were not correlated (Spearman *r* = 0.06, *p* = 0.37, *n* = 251). There were also no differences between the treatments in the number of attacks the helpers received from the breeders (square-root(attacks+3/8) transformed, GEE, effect of helper size: 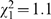, *p* = 0.31; treatment: 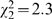, *p* = 0.32; interaction: 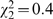, *p* = 0.82). In contrast, there was a positive correlation between submissive behaviours and the attacks the helpers received from the breeders (Spearman *r* = 0.17, *p* = 0.008, *n* = 251), but not between workload and attacks the helpers received from the breeders (Spearman *r* = –0.005, *p* = 0.94, *n* = 251). Therefore, we assessed whether the relative level of help and submission changed under the risk of predation by testing if the ratio of help to breeder attacks received (‘help ratio’) and the ratio of submission to breeder attacks received (‘submission ratio’) differed among the treatments. Only large helpers increased their help ratio under the risk of predation (Fig. 5a, GEE, effect of helper size: 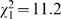, *p* = 0.001; treatment: 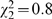, *p* = 0.66; interaction: 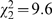, *p* = 0.008). No effects of the treatment on the submission ratio were discernible, but large helpers showed more submission to the breeders per aggression from the breeders received, than medium helpers did (Fig. 5b, GEE, effect of helper size: 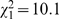, *p* = 0.001; treatment: 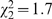, *p* = 0.42; interaction: 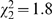, *p* = 0.40).

## Discussion

Predation risk is known to affect many aspects of animal behaviour, e.g. locomotion (e.g. [56], [57], vigilance (e.g. [58], [59]), shoaling (e.g. [60], [61]), courtship (e.g. [62]), the likelihood of engaging in territorial intrusions (e.g. [63]) and resource monopolisation (e.g. [64], [65]). In contrast, experimental studies on the effects of predators on helping behaviour have been limited (e.g. [66]) and this has been the focus of the present experiment. Previously we showed that in our experiment predation risk reduced (i) the likelihood of helpers breeding independently, (ii) helper and breeder survival and (iii) reproductive success of breeders [11]. Furthermore, we showed that helpers hid more and stayed closer to protective shelter under the risk of predation [11].

In this study we found a pronounced negative effect of predation risk on helper feeding, which was not only due to helpers hiding more, but also due to helpers staying closer to shelter where intake rates were lower, compared to the control treatment. We conclude that helpers might have needed to offset this reduced intake by saving energy in other behaviours, like helping and submissiveness [35], [36], making any negative effects on these behaviours due to predation risk difficult to interpret.

Despite helpers were feeding less, we detected no significant reductions in helping behaviours in the predation treatments compared to the control treatment, which refutes our hypothesis 3 (reproductive benefits/inheritance, Table 3). In contrast, an increase in territory defence (but only when corrected for the distance to the shelter effects) and territory maintenance (but only detected in the digging experiment) under the risk of predation is compatible with our hypothesis 1 ([67] pay-to-stay, Table 3). This hypothesis can also accommodate the fact that helpers defended the group against the introduced predator, something which we clearly did not expect if helpers were solely adjusting their behaviour to avoid risk (hypothesis 2). We found no evidence that predation risk affected the overall level of within-group conflicts, social contacts and breeder-helper punishment. Furthermore, helper submissiveness, but not helping behaviour, correlated with breeder punishment (the latter consistent with hypothesis 2). Nevertheless, large helpers increased their relative rate of helping to attacks received by the breeders in the predator treatments (Fig. 5), and large helpers were on average also more submissive to the breeders compared to medium helpers in all treatments. This suggests that large helpers, but not medium helpers, may have appeased dominants more under the risk of predation, compared to the control treatment, consistent with our hypothesis 1 (pay-to-stay). Note that under the risk avoidance hypothesis 2, we would expect the opposite pattern: medium helpers were under higher risk of predation compared to the large helpers [11], so medium helpers should be more willing to appease the dominants by submissive behaviour to ensure continued group membership under risk compared to the large helpers. Increases in helping behaviour in the predation treatments compared to the control treatment due to direct benefits (e.g. subordinate female reproduction [33], [37]–[38]) are unlikely to apply, e.g. due to the very low group reproductive success in groups under predation risk ([11]; contra our hypothesis 3, and contra the pay-to-reproduce hypothesis of [33]).

**Table 3:** Expected effects of the predator treatments under the three hypotheses mentioned in the introduction and the observed differences (-: predator treatments < control treatment, 0: predator treatments  =  control treatment, +: predator treatments > control treatment).

	Predicted by hypothesis			Observed
	Pay-to-stay	Risk avoidance	Reproductive benefits	
Territory maintenance	+	+	-	**0+** c
Territory defence	+	-	-	**+**
Predator defence^a^	+	-	-	**-+** d
Within-group aggression	-	e	+	**0**
Within-group contacts	+	e	-	**0**
Help ratio^b^	+	e	-	**+**
Submission ratio^b^	+	e	-	**0**

a If helpers showed any predator defence in the predator treatments.

b Help or submission as ratio of breeder punishment.

c No effect in the focal observations, but effect in digging experiment.

d Helpers joined defence against the introduced predator, but also clear evidence for risk avoidance due to aggression declining with *N. pulcher* body size.

e No clear predictions here, but there was a correlation between helper submissiveness and breeder punishment (supporting risk avoidance), which was not matched by an increase in the submission ratio in the predator treatments compared to the control treatment (contra risk avoidance).

Summarising, the majority of our results are compatible with the pay-to-stay hypothesis (Table 3, [23]–[27], [32], [67]). However, risk avoidance is also likely playing a role, particularly because aggression against the introduced predator strongly declined with decreasing group member body size (i.e. virtually zero for small and medium helpers, who are under highest risk [11]), and because of the patterns of joint predator defence (discussed in more detail below). Moreover, helpers stayed closer to shelter under the risk of predation (see also [11]), where food intake was lower, also suggesting risk avoidance. Finally, helpers increased their digging effort inside the breeding shelter under the risk of predation (when this shelter was experimentally covered with sand), which serves the breeding pair, but may also serve the helpers themselves as hiding shelters to decrease risk (although not all group members are allowed to hide there and most group members are not allowed there at all during reproduction; and group members preferably use their private shelter for hiding, own observations). We also predicted large helpers to react differently to the treatments than the medium helpers, which was confirmed by the help ratio (Fig. 5), but not by the absolute helping levels (Fig. 1, no significant interactions between treatment x helper size). Predators also had notable effects on spacing behaviour, thereby affecting some helper behaviours, discussed in more detail below.

### Predator effect on spacing and food intake

Helpers stayed closer to protective shelter under the risk of predation and had reduced feeding rates compared to the control treatment. All else being equal, reduced feeding is expected to reduce body mass accumulation (e.g. [68]), which will have profound effects on fitness [21], [33], [37]–[38]. Large helpers had a higher total food intake than medium helpers, because large helpers hid less and thus had more time available for feeding than medium helpers, despite medium helpers having a higher feeding rate than large helpers (corrected for the mean distance from the shelter). This confirms observations of feeding rates of *N. pulcher* in the north of Lake Tanganyika (Burundi), where with increasing body size individuals fed higher up in the water column (so further away from shelter), where food was more abundant [52].

Our results are in agreement with previous work on the relationships between predation risk, refuge use and feeding rate in a range of taxa (e.g. [69], [70]; see review [71]). For example, coral reef fish reduced their foraging time and hid more under the risk of predation [72]. Territorial brown trout *Salmo trutta* were willing to invest more in defending a high cover territory under the risk of predation [65]. We found no effect of the number of adults protecting the group against predators on the focal helper feeding rate, suggesting that feeding *N. pulcher* do not rely on vigilant group members to warn them from approaching predators. Consistent with this explanation is that we have never observed sentinel behaviour in foraging groups of *N. pulcher* (as occurs in carnivores, e.g. [73]; and birds, e.g. [74]), although helpers and breeders may be seen guarding fry feeding at the entrance of the breeding shelter, which is likely to enhance fry feeding rates.

Helper spacing behaviour also affected the frequencies of two helping behaviours. First, the frequency of territory maintenance (digging) was higher for helpers staying close to shelter. In retrospect, this effect could be expected, because only helpers visiting the shelters could engage in digging or carrying sand away from the shelters. Second, the frequency of territory defence was higher for helpers wandering further away from shelter. This effect might be partly due to helpers that wander away from the shelter encountering more often heterospecific and conspecific fish (e.g. members from neighbouring groups) compared to helpers that stay in or close to the shelters. Therefore, territory defence in cichlids might need to be interpreted with care in future studies, because it might include helpers exploring their (near) surroundings (e.g. to locate breeding vacancies or high quality feeding sites in the water column) and engage in aggressive interactions as a result.

### Defence against the introduced predators

Helpers engaged in risky territory defence against the introduced predators (see also [40], [75]). Interestingly, the per capita attack rate increased for all group members with the number of adults inside their group in the large predator treatment, but not in the medium predator treatment. In contrast, no effects of the number of adults on territory defence (excluding the introduced predator) were detected. When the risk of predation is high, group members apparently decide to attack more often if there is assistance from other group members, which may have two mutually non-exclusive adaptive explanations. It may (i) reduce the risk for the defender or (ii) cause a non-linear (e.g. exponential) increase of attack efficiency with an increasing number of defenders. Similar patterns of cooperative territory defence have been observed in other species. For instance, juvenile lions *Panthera leo* were more likely to join adult females in territory defence with increasing age and when the number of defending adults was high (or the number of intruders was low), i.e. when personal risk of defence was low [76]. Group mobbing of predators also occurs commonly in cooperatively breeding birds (e.g. [77], [78]), and results of a predator exclusion experiment in the bicolored wren *Campylorhynchus griseus* suggested that helpers assisting breeders in defending the nest from terrestrial predators may be critical for offspring survival [66]. Reproductive benefits from joint predator defence can also be found in less complex breeding associations, e.g. communally nesting female insects [79], [80]. Generally, it is unknown whether such synergistic effects result merely from an increase in defence frequency or from behavioural coordination of the defenders. This is also unknown for our study.

### Conclusions

According to the ‘pay-to-stay hypothesis’ [23], [24], cichlid dominant breeders should incur some costs from harbouring subordinate group members: in males due to paternity loss, particularly to large subordinate males [20]–[22]; and in females due to a reduction in growth rate [38], but not reproductive success [37], [38]. These costs should be somehow offset by the benefits subordinates provide to the dominants [4], [23], [24], for instance offspring survival [17], [18] and workload reduction [31] including parental care [33], [37]. However, without knowing the fitness benefits of each single helping behaviour to both the actor and the recipient, conclusions must remain preliminary (e.g. by manipulating investment in single helping behaviours only [26], combined with measuring fitness effects). In this study, we found predominantly evidence for the pay-to-stay hypothesis, and also for the risk avoidance hypothesis. Nevertheless, we suggest future studies should measure reproductive participation, survival and territory inheritance under varying risk of predation, taking refuge use into account [81]. Another way to proceed would be to manipulate the expected future benefits cichlid subordinates (e.g. [82], [83]) or dominants are likely to acquire from helping behaviour, and measure changes in helping behaviour and breeder-helper conflict in concert. Manipulations of dominant fitness would be particularly helpful to test the pay-to-stay hypothesis (e.g. reducing the dominant's clutch size suggesting egg eating by a subordinate [38]), as it should immediately lead to breeder punishment of subordinates [44], and in the most extreme case to dominants evicting subordinates from their group [20], [32].

## Supporting Information

Table S1 Focal behaviour: results of separate GEE base models (n = 251 for each model), testing for fixed effects of the treatment (df = 2), helper size (df = 1) and their interaction (df = 2) on focal helper feeding (normal distributions) and behaviour (poisson distributions: log-link). The models were repeated, GEEs with adults and distance, by adding the covariates number of adults inside the focal's group (>35 mm SL group members, df = 1) and the focal's mean distance spent from shelter (cm, ln-transformed, df = 1) to each respective base model. Significant p-values are indicated in bold.
(0.09 MB DOC)
