# Supplementary material for: Helper Response to Experimentally Manipulated Predation Risk in the Cooperatively Breeding Cichlid Neolamprologus pulcher
Source: PLoS One. 2010 May 26;5(5):e10784. doi: 10.1371/journal.pone.0010784 (PMC2877084; doi:10.1371/journal.pone.0010784)
Supplement: Table S1 — Focal behaviour: results of separate GEE base models (n = 251 for each model), testing for fixed effects of the treatment (df = 2), helper size (df = 1) and their interaction (df = 2) on focal helper feeding (normal distributions) and behaviour (poisson distributions: log-link). The models were repeated, GEEs with adults and distance, by adding the covariates number of adults inside the focal's group (>35 mm SL group members, df = 1) and the focal's mean distance spent from shelter (cm, ln-transformed, df = 1) to each respective base model. Significant p-values are indicated in bold. (0.09 MB DOC) [file pone.0010784.s001.doc]

**Supplementary Table S1 for** ***Helper Response to Experimentally Manipulated Predation Risk in the Cooperatively Breeding Cichlid Neolamprologus pulcher***

**Dik Heg, Michael Taborsky**

**Table S1.** Focal behaviour: results of separate GEE base models (*n* = 251 for each model), testing for fixed effects of the treatment (df = 2), helper size (df = 1) and their interaction (df = 2) on focal helper feeding (normal distributions) and behaviour (poisson distributions: log-link). The models were repeated, GEEs with adults and distance, by adding the covariates number of adults inside the focal’s group (>35 mm SL group members, df = 1) and the focal’s mean distance spent from shelter (cm, ln-transformed, df = 1) to each respective base model. Significant *p*-values are indicated in bold.

|  | GEE base models | |  | GEE with adults and distance | |
| --- | --- | --- | --- | --- | --- |
| *Dependent variable*, Independent variables | χ^2^ | *p* |  | χ^2^ | *p* |
| *Feeding rate / minute not hiding* | |  | | |  |
| Treatment | 13.5 | **0.001** |  | 0.7 | 0.71 |
| Helper size | 14.8 | **<0.001** |  | 12.5 | **<0.001** |
| Treatment*helper size | 2.0 | 0.37 |  | 3.5 | 0.17 |
| Number of adults |  |  |  | 0.1 | 0.82 |
| Distance |  |  |  | 57.6 | **<0.001** |
| *Total food intake* | |  | | |  |
| Treatment | 12.6 | **0.002** |  | 0.9 | 0.64 |
| Helper size | 5.2 | **0.022** |  | 17 | **<0.001** |
| Treatment*helper size | 1.3 | 0.51 |  | 2.9 | 0.24 |
| Number of adults |  |  |  | 0.1 | 0.8 |
| Distance |  |  |  | 41.6 | **<0.001** |
| *Digging* | |  | | |  |
| Treatment | 0.8 | 0.67 |  | 1.8 | 0.42 |
| Helper size | 0.5 | 0.47 |  | 2.5 | 0.11 |
| Treatment*helper size | 1.5 | 0.48 |  | 2.1 | 0.35 |
| Number of adults |  |  |  | 0.5 | 0.47 |
| Distance |  |  |  | 4.0 | **0.046** |
| *Territory defence*^*^ |  |  |  |  |  |
| Treatment | 2.2 | 0.33 |  | 6.8 | **0.033** |
| Helper size | 0.9 | 0.35 |  | 1.5 | 0.23 |
| Treatment*helper size | 1.2 | 0.56 |  | 0.4 | 0.83 |
| Number of adults |  |  |  | 0.4 | 0.52 |
| Distance |  |  |  | 22.5 | **<0.001** |
| *Within-group aggression* | |  |  |  |  |
| Treatment | 0.5 | 0.78 |  | 0.3 | 0.86 |
| Helper size | 1.7 | 0.19 |  | 3.1 | 0.081 |
| Treatment*helper size | 1.1 | 0.58 |  | 1.3 | 0.53 |
| Number of adults |  |  |  | 2.9 | 0.089 |
| Distance |  |  |  | 2.7 | 0.1 |
| *Within-group social contacts* | |  | | |  |
| Treatment | 3.6 | 0.17 |  | 2.3 | 0.31 |
| Helper size | 0.02 | 0.89 |  | 0.4 | 0.54 |
| Treatment*helper size | 0.9 | 0.63 |  | 0.6 | 0.75 |
| Number of adults |  |  |  | 1.8 | 0.17 |
| Distance |  |  |  | 2.5 | 0.12 |

Results were corrected for random group effects (repeated measures of 50 groups, entered as subjects in the GEEs); and time spent outside shelter (covariate) for digging, territory defence, within-group aggression and within-group social contacts. The scaling parameter was adjusted using the deviance method in each model

^*^ Excluding defence against the introduced predator
